# Direct imaging of molecular symmetry by coherent anti-stokes Raman scattering

**DOI:** 10.1038/ncomms11562

**Published:** 2016-05-18

**Authors:** Carsten Cleff, Alicja Gasecka, Patrick Ferrand, Hervé Rigneault, Sophie Brasselet, Julien Duboisset

**Affiliations:** 1 Aix-Marseille Université, CNRS, Centrale Marseille, Institut Fresnel, UMR 7249, Domaine Universitaire de Saint Jérôme, Avenue Escadrille Normandie Niemen, Marseille, F-13397, France; 2 Quebec Mental Health Institute Research Center, Laval University, Québec, Quebec, Canada G1J 2G3; 3 Centre d'Optique, Photonique et Laser (COPL), Laval University, Québec, Quebec, Canada G1V 0A6

## Abstract

Nonlinear optical methods, such as coherent anti-Stokes Raman scattering and stimulated Raman scattering, are able to perform label-free imaging, with chemical bonds specificity. Here we demonstrate that the use of circularly polarized light allows to retrieve not only the chemical nature but also the symmetry of the probed sample, in a single measurement. Our symmetry-resolved scheme offers simple access to the local organization of vibrational bonds and as a result provides enhanced image contrast for anisotropic samples, as well as an improved chemical selectivity. We quantify the local organization of vibrational bonds on crystalline and biological samples, thus providing information not accessible by spontaneous Raman and stimulated Raman scattering techniques. This work stands for a symmetry-resolved contrast in vibrational microscopy, with potential application in biological diagnostic.

## Abstract (web summary)

Coherent Raman imaging is a high fidelity technique to obtain chemical-sensitive images, however sub-diffraction molecular organization information is still missing. Here, the authors exploit molecular bond symmetries to access the microscopic organization of molecules in a single image acquisition.

## Graphical abstract

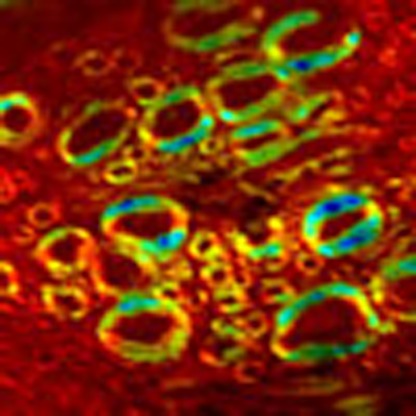

Obtaining information on the organization of matter on the micrometer-scale with non-destructive methods still remains a challenge in chemical physics and biology. One well-established method for extracting matter organization information is fluorescence microscopy, which uses fluorescent molecules or proteins to tag the sample. However, this technique is limited to the observation of the probe itself, which may differ from the sample organization. Coherent Raman scattering (CRS) microscopy technique has proven to be powerful due to its label-free, three-dimensional, chemical selective and real-time imaging capabilities^1^,^2^,^3^,^4^,^5^,^6^. In coherent anti-Stokes Raman scattering (CARS), two beams of different frequencies interact with the sample to excite a vibrational resonance. A probe beam is used to probe the vibrational excitation by generating a new anti-Stokes frequency shifted beam. Unfortunately, CARS is limited by a non-resonant four wave mixing (FWM) background resulting in reduced image contrast and chemical selectivity. Recently, it has been shown that stimulated Raman scattering (SRS) can provide vibrational spectra without non-resonant background allowing to report vibrational information with high fidelity and high efficiency^7^,^8^,^9^,^10^ and CARS can benefit from non-resonant background to heterodyne-amplify weak Raman signals^11^. It is well-known that the symmetry properties of matter have a strong influence on its physical properties, for example, in crystalline samples. Similarly, it has been found that in biological environments anisotropic and symmetry properties of tissue are often related to specialized biological functionality. Nevertheless, the organization of molecular bonds in the focal volume, which is of particular interest in numerous situations where the medium is organized, is not contained in the spectral information. However, seminal works from nonlinear optics pioneers have shown that the Cartesian components of the nonlinear susceptibility tensor 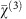 express the vibrational symmetry properties^12^,^13^. To read the tensor elements, polarization-resolved schemes have been proposed decades ago^14^, stimulating more recent developments in microscopy^15^,^16^,^17^,^18^,^19^,^20^. The molecular organization from a sample is usually retrieved by acquiring a stack of images from different polarization angles of the excitation or detection light fields, requiring long acquisition times and time consuming post-processing^21^.

In this article, we introduce a label-free microscopy technique that is able to retrieve the individual symmetry orders of molecular organization in a single image acquisition. The symmetry-resolved CARS (SR-CARS) signal not only depends on the presence of molecular bonds, but also on their organization within the focal volume. By switching between combination of left- and right-handed circular polarization states for the involved fields, it is possible to directly image individual symmetry contributions of the sample. Beyond this organization selectivity, we show that our technique can (1) suppress the isotropic background in CARS images and spectra, thus enhancing the contrast by 1–2 orders of magnitude, and (2) retrieve quantitative information without pre-knowledge information on the molecular organization, without post-processing and independently of sample orientation in the transverse plane. SR-CARS provides higher chemical selectivity based on different symmetry characteristics, which are not accessible with regular spontaneous Raman or SRS microscopy.

## Results

### Light matter symmetry matching

A direct read-out of a specific sample symmetry is possible when the light field tensor probing the sample only consists of the targeted symmetry of the sample^22^,^23^. In practice, the detected CARS electric field amplitude *E*_as_ (anti-Stokes) along a specific polarization direction is the projection of a tensor 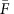, representing all involved light fields, onto the 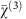 susceptibility tensor of the medium


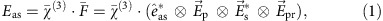


where ⊗ is the dyadic product and * stands for the complex conjugate, 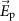, 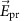 and 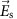 are the pump, probe and Stokes fields, respectively, and 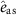 is the unit vector along the polarization direction of the emitted anti-Stokes field, such that the four vector fields create a rank-four tensor^24^.

As our technique targets the investigation of sample's symmetry, it is convenient to choose spherical coordinates 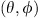, for which left- 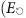) and right-handed 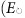) circular polarization and linear *z*-polarization states (*E*_*z*→_) can be described by a set of spherical harmonic functions 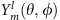 with *l*=1


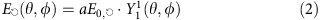



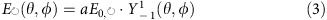



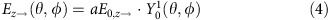


where 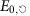, 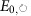, *E*_0,→_ are the electric field amplitudes associated to the quantum number *m*=1, *m*=−1 and *m*=0, respectively and 
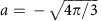
 is a normalization factor (see Supplementary Note 1).

The advantage of using spherical harmonic functions is that an *m*-value is a direct reporter of the presence of an *m*-folded rotational invariant symmetry in the sample plane. In case of the CARS field tensor 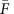 in equation (1), the multiplication of spherical harmonic functions generates a new set of spherical harmonic functions of fixed *m*-values, allowing to read *m*-values higher than the initial individual light fields and to determine the symmetry of the combined light fields


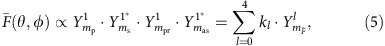


with the resulting 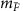-value being a summation of the field's *m*-values


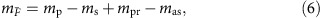


where *k*_*l*_ are the Clebsch–Gordan coefficients weighting the different spherical harmonic functions (see Supplementary Note 1) and 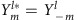. Therefore, the light field tensor 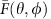 has a 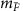-rotational symmetry in the sample plane defined by the *m*-values of the individual incident and emitted light fields, see equation (6).

When 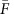 (light) probes 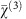 (matter) in a CARS process (equation (1)), it can be seen, from the orthogonality of spherical harmonic functions, that 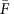 only probes parts of the 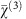-tensor with identical rotational invariant symmetries (that is, identical 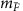). Thus, engineering of 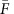 allows the direct read-out of specific sample symmetries, creating a symmetry-based contrast mechanism.

**Figure 1:**
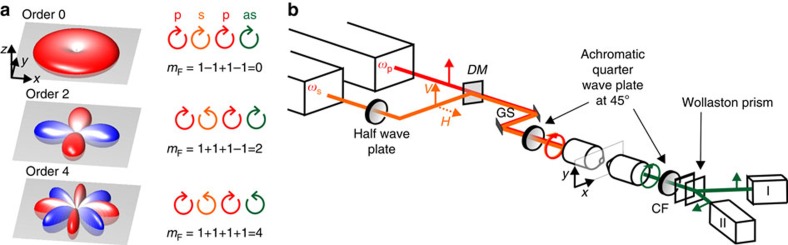
Symmetry-resolved CARS setup. (**a**) Generated CARS light field 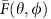 with symmetry orders 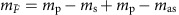 resulting from the combination of circular polarization states for the pump (red), Stokes (orange) and detected CARS (green) fields; (**b**) schematic of the experimental setup (CF, CARS filter; DM, dichroic mirror; GS, galvo-scanners), switching the Stokes field between *V*- and *H*-polarization states before the first quarter-wave plate allows switching between Stokes field 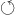
- and 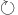-polarization at the sample plane. Using two photomultiplier tubes (I/II) detecting 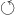
- and 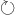-polarized CARS fields allows to target the different symmetry orders 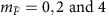.

Figure 1a displays the different light fields 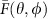 of symmetry orders 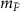 that can be generated using circularly polarized light and degenerated CARS (*m*_p_=*m*_pr_). As circular polarizations provide only *m*=1 or *m*=−1 values, sample rotational symmetry contribution of order 0 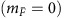, order 2 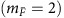 and order 4 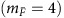 can be specifically addressed. Using two detectors for the emitted 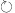 and 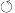 CARS polarization states allows the independent and simultaneous detection of symmetry contributions of order 0 (isotropic) and 2 (twofold symmetry), respectively, if the pump, Stokes and probe beams have the same circular polarization states (Fig. 1b). If the Stokes circular polarization state is changed from 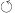 to 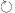, symmetries of order 2 and 4 (fourfold) are then probed independently and simultaneously. Using circularly polarized light at both excitation and detection paths in CARS then creates a new symmetry-resolved imaging modality that has the unique ability to extract local orientational symmetry information from any sample. The orders 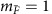 and 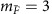 are in principle also accessible, but require excitation or detection of *z*-polarization contributions, which is not considered in this work. Note that since the obtained information is invariant on rotation around the *z*-axis, each symmetry order image does not depend on the sample orientation in the sample plane.

### Multi lamellar vesicles

**Figure 2:**
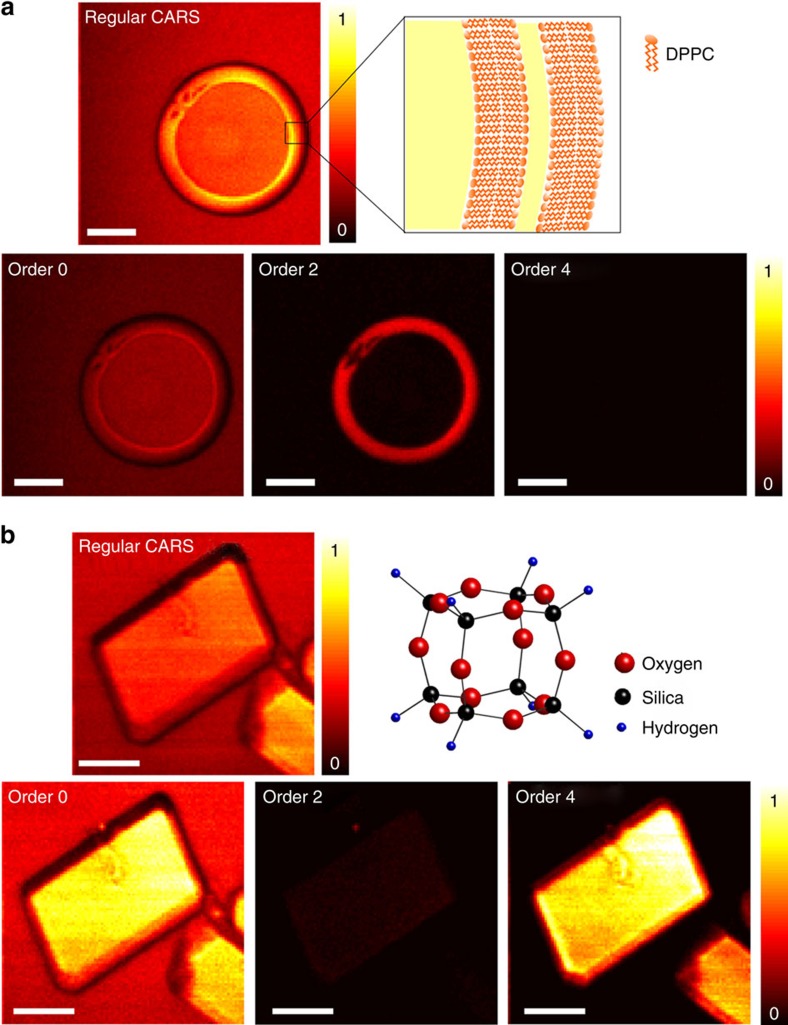
Symmetry-resolved CARS imaging. (**a**) multi lamellar DPPC vesicle (MLV) imaged with Regular and SR-CARS at 1,133 cm^−1^. (**b**) HT8 zeolite crystal imaged with Regular and SR-CARS at 932 cm^−1^. Schematic representation of the membrane structure of a MLV and the cubic unit cell of HT8 zeolite are also shown. Scale bar, 10 μm.

To demonstrate the potential of SR-CARS imaging, we applied our scheme to model samples of known symmetry. 1,2-Dipalmitoylphosphatidylcholine (DPPC) multi lamellar lipid vesicles (MLVs) are made of a tight packing of lipid layers, which form a ring of highly ordered matter with twofolded symmetry (see drawing in Fig. 2) and a lipid orientational distribution close to a Gaussian angular shape^20^. Regular CARS imaging of MLVs on C–C stretching vibration at 1,133 cm^−1^ embedded in water, shows a poorly contrasted image due to the non-resonant background, see Fig. 2a. Using SR-CARS imaging, different symmetry contribution can be separately imaged, namely the isotropic 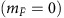, twofold 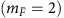 and fourfold 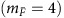 contributions of the equatorial section of the MLV in the sample plane. Figure 2a shows that the aqueous solution surrounding the MLV is only visible in the order 0 image, due to its purely isotropic nature. The microscopic organization of lipids results in a strong order 2 signal coming from both nonresonant and resonant contributions, allowing background-free imaging of the MLV with a clearly superior contrast respect to regular CARS image. At last, no visible order 4 is present at this frequency, meaning that the lipid C–C stretching vibration at 1,133 cm^−1^ is symmetric, in contrast to anti-symmetric vibrations (see Supplementary Fig. 1). To obtain more quantitative information about the molecular organization, the order 2 image is normalized by the total intensity image (sum over all the order images) pixel by pixel and square rooted, leading to a direct read-out of the order 2 susceptibility tensor contribution. This normalized order 2 is found to be ∼0.5 (see Supplementary Fig. 2) and is in good agreement with previous results obtained in similar systems^20^.

### Zeolite crystal

Since molecular vibrational resonances belong to a variety of symmetry groups, SR-CARS can be used to retrieve the vibration symmetries. As a second model system we selected the cubic crystal octahydrosilasesquioxane H_8_Si_8_O_12_ crystal (HT8) zeolite, which forms microscopic scale crystals belonging to the *O*_*h*_ crystalline point group, and has a fourfold symmetry for the 932 cm^−1^ O–Si–H vibrational resonance addressed here^25^. Regular CARS imaging of such a crystal embedded in water shows a poorly contrasted image (Fig. 2b) due to the nonresonant background. Employing SR-CARS, the surrounding background disappears in the order 4 image, enhancing the CARS image contrast of the crystal with respect to its isotropic surrounding by a factor of 100. The order 2 image, as expected from a pure fourfold symmetry, shows no visible signal.

**Figure 3:**
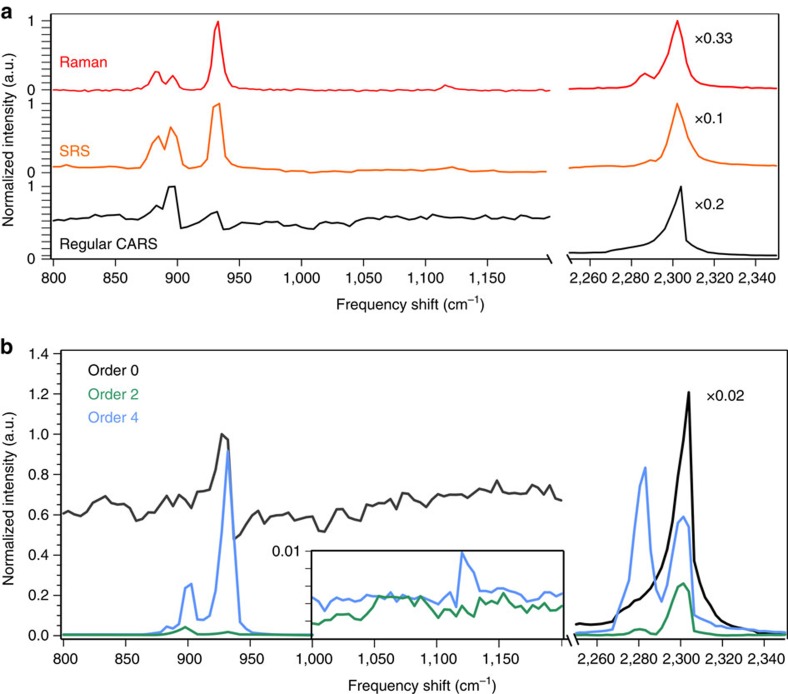
Symmetry-resolved CARS spectrum. (**a**) Vibrational spectra of a HT8 zeolite crystal obtained with spontaneous Raman, SRS and regular CARS. (**b**) SR-CARS order 0, 2 and 4.

SR-CARS can be used to increase spectral contrast and separate close-by or overlapping resonances. Figure 3a shows the vibrational spectrum (obtained by scanning the Stokes beam wavelength) of a HT8 zeolite crystal acquired with spontaneous Raman scattering, SRS and regular CARS. In comparison to spontaneous Raman and SRS spectra, regular CARS is clearly affected by a nonresonant background generated by the crystal itself, which prevents from visualizing all the resonances of HT8 in the scanned spectral range.

Figure 3b displays order 0, order 2 and order 4 spectra acquired with the SR-CARS scheme. The first observation is that order 4 provides a highly contrasted background-free spectrum. It reveals in particular a weak vibrational resonance at 1,120 cm^−1^ that is invisible in regular CARS. Moreover, it clearly separates resonances that are less distinct in Raman or SRS (∼2,300 cm^−1^). The separation of overlapping vibrational resonances with SR-CARS is possible, as the resonances are of different vibrational mode types with different symmetry properties. As a result, they have similar amplitudes for their fourfolded symmetry contributions (thus allowing to separate them) while the total amplitudes of the two resonances are of different orders of magnitude such that they can hardly be distinguished in spontaneous Raman or SRS. While regular CARS of order 0 spectra are clearly distorted by the intrinsic interference with the nonresonant background (which is itself primarily of order 0), the high order symmetry filtering extracts much clearer spectral information (see also the whole spectral scan in Supplementary Movie 1). The order 2 spectrum shows weak signals, which we attribute to residual polarization leakage. The second observation is that all the resonances of the crystal do not have the same symmetry-resolved spectra. The *A*_1*g*_ resonance at 2,302 cm^−1^ has mainly order 0 contribution, the order 4 is <1% of the isotropic order and comes from optical leakage. The *T*_2*g*_ resonances at 883 cm^−1^, 897 cm^−1^, 1,117 cm^−1^, 2,286 cm^−1^ have only a strong order 4 contribution and the *E*_*g*_ resonance at 932 cm^−1^ has both order 0 and order 4 contributions. The decomposition of each mode on the symmetry orders is determined by the selection rules and detailed in Supplementary Fig. 3 and Supplementary Note 2. The three different modes of zeolite investigated here have three different signatures on the SR-CARS spectra, allowing an unambiguous experimental identification of each mode.

### Myelin sheaths

We now focus on myelin sheaths, that are highly organized multilayer membranes made of lipids and proteins surrounding the axon of neurons. Myelin integrity is essential for the propagation of action potential and CARS has proven to be a powerful label-free technique for myelin imaging, targeting lipid vibrational bonds^26^,^27^. Polarization-resolved CARS has been reported to describe myelin molecular organization^20^, and to relate this organization to demyelination processes^28^, however, such experiments take minutes as they require multiple images with different incoming linear polarization states. Here we apply SR-CARS imaging to mice spinal cords and show that it can enhance information and reveal the full molecular lipid organization in a single image acquisition, within biological tissues.

**Figure 4:**
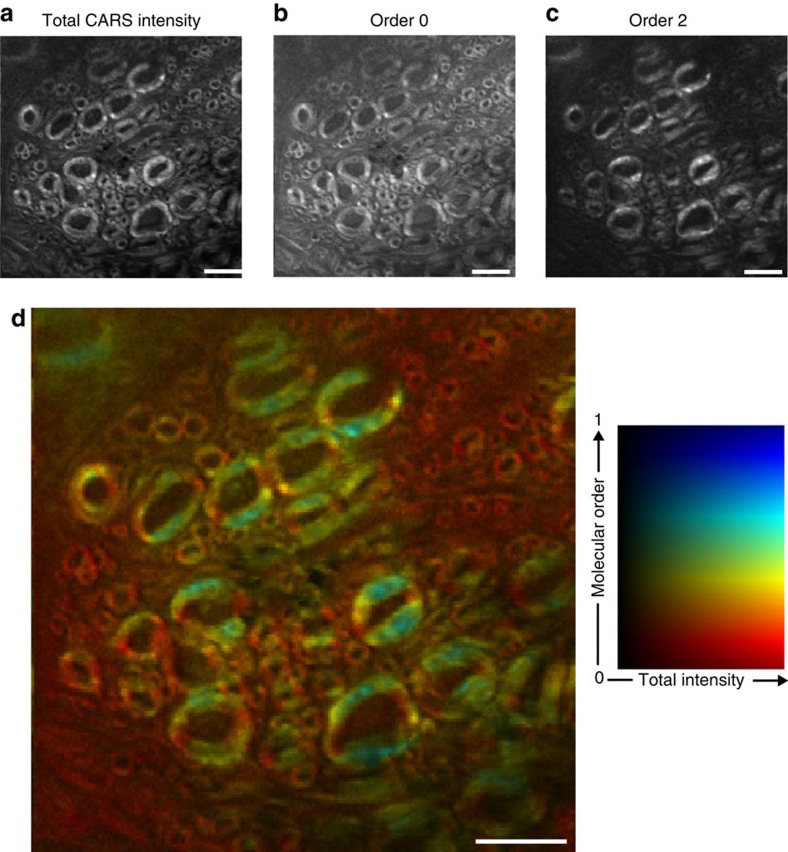
Symmetry-resolved imaging of *ex vivo* myelin sheaths in mice spinal cord. (**a**) Total CARS intensity (circular excitation and unpolarized detection). (**b**) SR-CARS image of order 0, exhibiting isotropic symmetry contributions in the sample. (**c**) SR-CARS image of order 2, highlighting the twofold symmetry CARS contribution associated to organized lipids. (**d**) Quantification of local molecular order in the myelin sheath using normalized order 2 image with respect to the total intensity image, pixel by pixel. The local molecular order is colour coded, from 0 (for completely disordered) to 1 (for fully oriented), while the total intensity is encoded in the pixel brightness. Scale bar, 5 μm. All data are representative of at least three independant experiments.

Figure 4a shows a CARS intensity image of an *ex vivo* mouse spinal cord transverse section, using circular excitation on the C–C vibrational bond at 1,099 cm^−1^. The vibration is chosen for its weak efficiency, as compared with CH_2_ stretching modes, to demonstrate the performance of SR-CARS scheme. The order 0 image (Fig. 4b), shows weak contrast due to the presence of lipids bonds surrounding the myelin sheath and an isotropic contribution of the nonresonant background. On the contrary, the order 2 image (Fig. 4c) highlights the ring-shaped myelin around the axons, exhibiting the fact that lipids are highly organized and oriented in such structures. The order 4 image shows very weak signals (not shown), similarly as in MLVs. To provide a quantitative information of molecular order in myelin sheath using SR-CARS, the order 2 image is normalized by the total intensity image (sum of all orders) pixel by pixel, to cancel the molecular density dependency, see Fig. 4d. The molecular order of lipids in myelin sheaths is visibly not homogeneously distributed, with highly organized regions like in MLV can be found (high order 2 values), as well as highly disordered regions with low order 2 (Fig. 2a). This heterogeneity can be attributed to different morphologies and molecular compositions present in multilayers surrounding axons, which is a topic of current interest to address possible molecular-level neurobiology diagnostics^28^. The symmetry-resolved modality developed here is then able, in a single image allowing for high-speed imaging, to reveal local heterogeneities that are absent from the regular CARS images.

## Discussion

This work presents a CARS imaging modality based on symmetry-selectivity, with unprecedented ability to enhance vibrational contrast and to reveal molecular bonds organization symmetry. The use of circular polarization makes this imaging modality independent of the sample orientation in the transverse sample plane, making this contrast enhancement efficient without the need to find an optimal polarization coupling direction. However, the use of polarization makes SR-CARS modality impossible within materials where polarization of light can not be maintained efficiently, such as birefringent materials or scattering media.

We exploited the specific symmetry of molecular bonds assemblies to strongly enhance the CARS image contrast in crystalline and tissue sample. In the past, many other techniques have been presented to improve contrast in CARS, though they mostly aimed to reduce the nonresonant background. A circular excitation approach was already developed in CARS by Upputuri *et al.*^17^ to remove the isotropic background and enhance the signal from anisotropic materials. Our SR-CARS scheme not only enhances the non isotropic CARS signal, it also brings additional key information such as the vibration symmetry and the symmetry of the probed molecular assemblies. Such symmetry retrieval is possible because SR-CARS is based on a theoretical background using the irreducible spherical formalism.

Finally, molecular orientational organization imaging can be exploited to achieve a structural contrast in biological samples, which can improve monitoring of biological processes and diseases, as well as diagnostics. This provides quantitative molecular bond symmetry imaging in a single image acquisition without the need for any polarizer rotation nor signal processing. Biochemical studies have shown that molecular organization in myelin sheaths is indeed highly affected in diseases like multiple sclerosis and leukodystrophies; however, no current imaging technique can quantitatively address this issue *in vivo*. Investigating biological functions related to molecular organization in real time would be highly valuable and SR-CARS could be the basis of high-speed diagnostics.

## Methods

### CARS microscope

CARS imaging was performed on a custom-built setup incorporating a picosecond stimulated Raman optical source^29^. This source is composed of two optical parametric oscillators (OPO1 and OPO2, Emerald, APE) synchronously pumped by a mode-lock frequency doubled Nd:YVO Laser (PicoTrain, HighQLaser) operating at 532 nm. The two mode-locked beams from OPO1 (pump) and OPO2 (Stokes) (pulse duration 5 ps, repetition rate 76 MHz) are overlapped in time and space and sent into a custom made scanning microscope. For spectral scan, the pump wavelength is fixed to 730 nm and the stokes wavelength is scanned from 775 to 790 nm and 870 to 885 nm. SR-CARS signals (order 0, 2 and 4) are detected in the forward direction using PMTs (Hamamatsu, H10682) working in photon-counting regime. Excitation and collection are provided by an numerical aperture=0.6 objective (Olympus UCPlan FL × 40). Incident powers at the sample plane were 1–4 mW for the pump beam and 1–5 mW for the Stokes beam depending on the samples. Imaging is performed by scanning galvanometric mirrors (typically, pixel dwell time of 50 μs, 100 × 100 pixels and scan range of 30 μm). A dedicated software controls the galvo mirrors, the acquisition card and the OPO wavelength tuning for spectral acquisition^30^.

To achieve SR-CARS imaging, an achromatic quarter-wave plate was inserted before the focusing microscope objective to excite the sample with circularly polarized light. The linear polarization state (before the quarter-wave plate) of the Stokes beam was switched between V- and H-polarization, resulting in a switching between 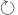 and 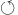-circular polarization states in the sample plane. The generated CARS signal passed through a second achromatic quarter-wave plate converting circular polarization to linear polarization. Subsequently, a Wollaston prism split the CARS beam into V- and H-polarization, which were detected individually with photomultiplier tubes. Consequently, the two photomultiplier tubes were sensitive to 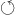 and 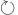-circular polarization states in the sample plane. To ensure that any desired circular polarization state of the excitation is properly delivered to the sample, we used a polarimeter described in ref. 31.

In the SRS operation mode, the pump beam was modulated in amplitude at a frequency of 20 MHz by an acousto-optic modulator (AOM—AAOptoelectronic MT200 A0,2-800). The stimulated Raman gain induced on the Stokes beam was detected in the forward direction by means of a high-speed photodiode and a fast lock-in amplifier (manufactured by APE) in a way similar to ref. 7.

The Raman spectrum was acquired using a HeNe laser at 632.8 nm and a spectrograph (Horiba iHR320) equipped with a Peltier-cooled CCD detector.

### MLV samples

MLVs were made from chain 1,2-dipalmitoylphosphatidylcholine (DPPC) lipids and 5% cholesterol. DPPC was hydrated in phosphate-buffered saline (pH 7.4) above the main phase-transition temperature (45 °C) for 1 h, leading to MLVs of 1–30 μm size in a solution enclosed between two spaced coverslips. The MLVs form almost spherical objects made of concentric multilayers of lipids. The images were performed at the equatorial plane of these objects where the distribution of the lipids is expected to be lying along the transverse sample plane.

### Zeolite samples

The sample studied in this work is an octahydrosilasesquioxane HT8 crystal, that has cubic symmetry and belongs to the O_*h*_ crystallographic point group. The HT8 crystal synthesis can be found in ref. 25. For CARS imaging, micrometric to millimetric size crystals are directly deposited onto a microscope coverslip and surrounded by water.

### Myelin samples

All experimental procedures have been performed in accordance with guidelines from the Canadian Council on Animal Care. Mice were anesthetized and perfused intracardially with 4% paraformaldehyde (PFA, Fischer Scientific, Pittsburgh) in 0.1 M phosphate buffer (PB). The whole spinal cord was dissected out from each mouse and placed in 4% PFA overnight. Lumbar spinal segments corresponding to L3–5 level were isolated from the spinal cord, rinsed several times with 0.1 M PB. About 30-μm-thick slices were cut in the sagittal plane using a vibratome (Leica, VT 1000).

### Data availability

We declare that the data supporting the findings of this study are available within the article and its Supplementary Information Files.

## Additional information

**How to cite this article:** Cleff, C. *et al.* Direct imaging of molecular symmetry by coherent anti-stokes Raman scattering. *Nat. Commun.* 7:11562 doi: 10.1038/ncomms11562 (2016).

## Supplementary Material

Supplementary Information Supplementary Figures 1-3 and Supplementary Notes 1-2

Supplementary Movie 1 Movie of regular CARS, order 0 and order 4 spectra. 88 images are taken while the Raman frequency was scanned from 778 to 1200 cm^-1^ and 36 images are taken from 2257 to 2347 cm^-1^. Scale bar 10 μm.
