# Supplementary material for: Direct imaging of molecular symmetry by coherent anti-stokes Raman scattering
Source: Nat Commun. 2016 May 18;7:11562. doi: 10.1038/ncomms11562 (PMC4873966; doi:10.1038/ncomms11562)
Supplement: Supplementary Information — Supplementary Figures 1-3 and Supplementary Notes 1-2 [file ncomms11562-s1.pdf]

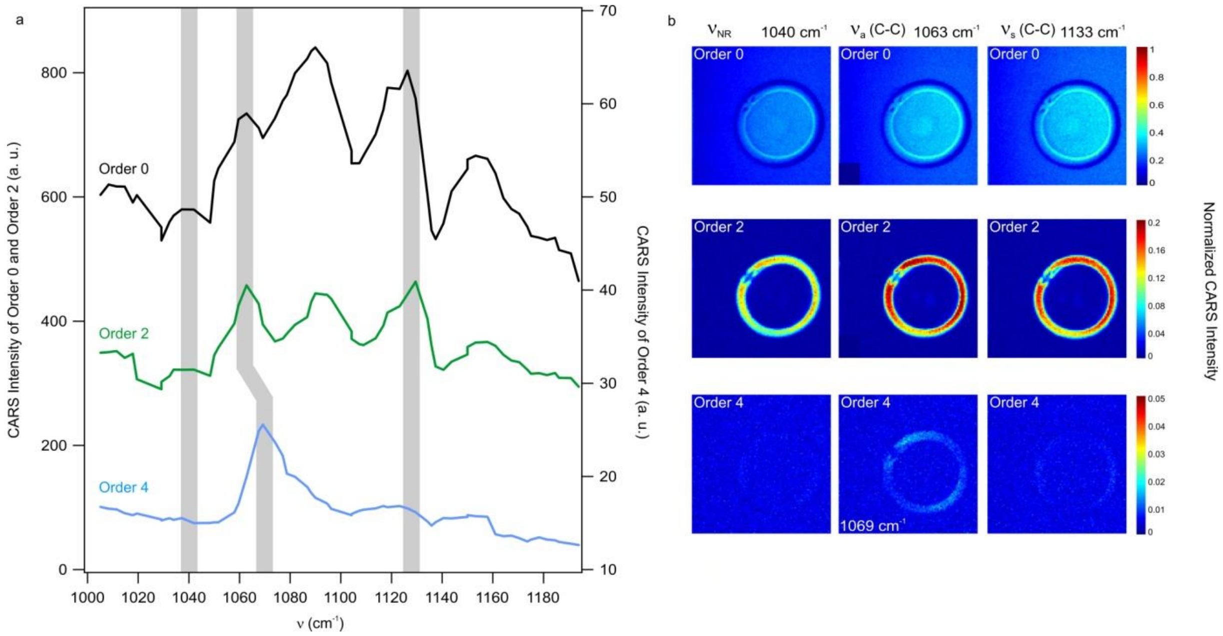

Supplementary Figure 1: **Symmetry-resolved CARS on multilamellar vesicle made of Dipalmitoylphosphatidylcholine (DPPC).** (**a**) SR-CARS spectrum. (**b**) SR-CARS images of MLV at 1040 cm-1 (non resonant), at 1063/1069 cm^{-1} (anti-symmetric stretching mode of C-C) and at 1133 cm^{-1} (symmetric stretching mode of C-C). The DPPC is known to have several Carbone-Carbone stretching modes in the 1000-1200 cm^{-1} region. The symmetric vibration at 1133 cm^{-1} has order 0 and order 2 contribution, whereas the anti-symmetric vibration at 1063 cm^{-1} has, in addition, an order 4 contribution. This contribution appears at 1069 cm^{-1} due to the absence of Fano effect with the non resonant background. For the same reason as zeolite, the order 4 in CARS comes from an order 2 in spontaneous Raman, which is the direct signature of a vibrational anisotropy.

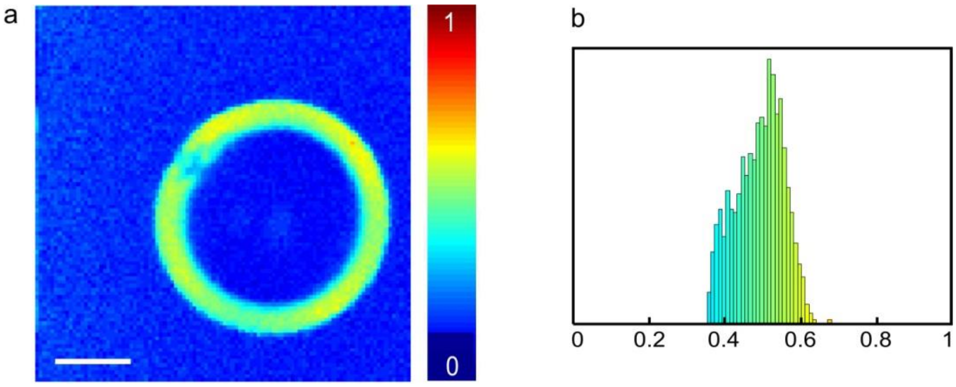

Supplementary Figure 2: quantitative molecular order on multi lamellar vesicle. (**a**) Square root of the ratio between order 2 and the total CARS intensity. (**b**) Histogram of the ratio values. Following the CARS signal symmetry decomposition, the order 0 and order 2 images are weighted by the molecular density. In order to retrieve quantitative information about the molecular organization independently from this density, the order 2 image needs to be divided by the total intensity image. The square root of this ratio allows to scale with a normalized susceptibility, independent of the molecular density. The average value of normalized order 2 on the MLV contour is 0.49 with a standard deviation of 0.06.

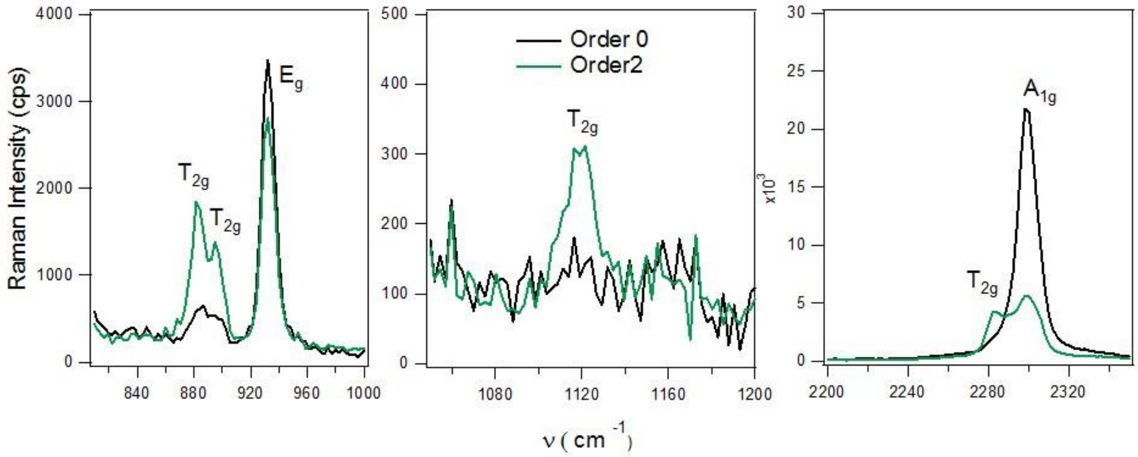

Supplementary Figure 3: Symmetry-resolved spontaneous Raman spectrum. The order 0 spectrum was recovered using left-hand circular polarization excitation and left-hand circular polarization detection. The order 2 spectrum was recovered using the same excitation and with a right-hand circular polarization.

Supplementary Note 1: Connection between circular polarization and spherical harmonics

Vectors quantities (rank 1 tensor) can be expressed either in the Cartesian basis *x,y,z* or in spherical basis $Y_m^{l=1}$ with m = -1, 0, 1. The electric fields expressed in the spherical basis write:

$$E_1^1 = -\frac{E_{0,\circlearrowleft}}{\sqrt{2}}(x+iy)$$

$$\boldsymbol{E_0^1} = \boldsymbol{E_{0,\nearrow}}\,\boldsymbol{z} \qquad (1)$$

$$E_{-1}^1 = \frac{E_{0,\circlearrowright}}{\sqrt{2}}(x-iy),$$

where $E_{0,\circlearrowleft}$, $E_{0,\nearrow}$ and $E_{0,\circlearrowright}$ represent the norms of the electric field. These field vectors components coincide with the in-plane left circularly polarized field, axial and right circularly polarized fields, which are all invariant through rotations around z axis. In the spherical basis, these three polarization states can be written in terms of $Y_m^{l=1}$ with m=1,0,-1.

$$E_1^1 = E_{\circlearrowleft} = -\sqrt{4\pi/3}\,E_{0,\circlearrowleft}\,Y_1^1$$

$$E_0^1 = E_{\nearrow} = -\sqrt{4\pi/3}\,E_{0,\nearrow}\,Y_0^1 \qquad (2)$$

$$E_{-1}^1 = E_{\circlearrowright} = -\sqrt{4\pi/3}\,E_{0,\circlearrowright}\,Y_{-1}^1$$

with

$$Y_1^1(\theta,\varphi) = -\sqrt{3/8\pi}\,sin\theta e^{i\varphi}$$

$$Y_0^1(\theta,\varphi) = -\sqrt{3/4\pi}\,cos\theta \qquad (3)$$

$$Y_{-1}^1(\theta,\varphi) = \sqrt{3/8\pi}\,sin\theta e^{-i\varphi}.$$

Consequently, the product of four electric fields involved in the constitutive equation

$$E_{as} = \bar{\chi}^{(3)}.\left(\hat{e}^*{}_{as}\otimes\vec{E}_p\otimes\vec{E}^*{}_s\otimes\vec{E}_{pr}\right), \qquad (4)$$

generates a field function $\bar{F}$

$$\bar{F} = \hat{e}^*{}_{as}\otimes\vec{E}_p\otimes\vec{E}^*{}_s\otimes\vec{E}_{pr}. \qquad (5)$$

In the case of degenerated CARS, when pump and probe fields are equal, and considering in-plane circular polarization states only, the field function $\bar{F}$ can only exhibit rotational invariant symmetries of order 0, 2 or 4

$$F_{m_{\bar{F}}=0}(\theta,\varphi) = E_1^1 E_1^{1^*} E_1^1 E_1^{1^*} = E_1^1 E_{-1}^1 E_1^1 E_{-1}^1 \propto \sin\theta^4$$

$$F_{m_{\bar{F}}=2}(\theta,\varphi) = E_1^1 E_1^{1^*} E_1^1 E_{-1}^{1}{}^* = E_1^1 E_{-1}^1 E_1^1 E_1^1 \propto \sin\theta^4\, e^{2i\varphi} \qquad (6)$$

$F_{m_{\bar{F}}=4}(\theta,\varphi) = E_1^1 {E_{-1}^1}^* E_1^1 {E_{-1}^1}^* = E_1^1 E_1^1 E_1^1 E_1^1 \propto \sin\theta^4\, e^{4i\varphi}$

The spherical functions $F_{m_{\bar{F}}}$ are linked to the spherical harmonic function by the Clebch-Gordan coefficient:

Order 0: $F_{m_{\bar{F}}=0} = \frac{1}{\sqrt{70}} Y_0^4 - \frac{2}{\sqrt{63}} Y_0^2 + \frac{2}{\sqrt{45}} Y_0^0$

Order 2: $F_{m_{\bar{F}}=2} = \frac{1}{\sqrt{28}} Y_2^4 - \sqrt{\frac{3}{14}} Y_2^2$ (7)

Order 4: $F_{m_{\bar{F}}=4} = Y_4^4$

Supplementary Note 2: relation between vibration symmetry and measured orders

The spontaneous Raman active vibration modes of the cubic space group can be decomposed on the spherical harmonic basis, based on the irreducible representation

A_{1g} → $\bar{\alpha}_{m_F=0}$

E_g → $\begin{cases}\bar{\alpha}_{m_F=0}\\ \bar{\alpha}_{m_F=2}\end{cases}$ (8)

T_{2g} → $\bar{\alpha}_{m_F=2}$

The Symmetry-resolved Raman spectrum shows the different symmetry orders of each resonance, see Supplementary Figure 1. The A_{1g} resonance is mainly present on the order 0, the T_{2g} is only present on the order 2 and the E_g resonance is present on both order 0 and 2. The nonlinear susceptibility $\chi^{(3)}$involved in the CARS process can be written as a tensorial product of the Stokes susceptibility by the anti-Stokes susceptibility

$$\bar{\chi}^{(3)} = \bar{\alpha}_{Stokes} \otimes \bar{\alpha}_{anti-Stokes}. \quad (9)$$

Using the spherical harmonic composition, the vibrational modes in CARS are decomposed along the orders

A_{1g} → $\bar{\chi}_{m_F=0}$

E_g → $\begin{cases}\bar{\chi}_{m_F=0}\\ \bar{\chi}_{m_F=4}\end{cases}$ (10)

T_{2g} → $\bar{\chi}_{m_F=4}$
